# The Effects of Carbonate on *Candida albicans* Filamentation, Biofilm Formation, and Antifungal Resistance

**DOI:** 10.1002/mbo3.70008

**Published:** 2024-11-13

**Authors:** Trenton P. Miedema, Kayla E. Grooters, Ian A. Cleary

**Affiliations:** ^1^ Department of Biomedical Sciences Grand Valley State University Allendale Michigan USA; ^2^ Department of Medicine Western Michigan University Homer Stryker M.D. School of Medicine Kalamazoo Michigan USA

**Keywords:** antifungals, *Candida albicans*, carbonate, filamentation

## Abstract

*Candida albicans*, a member of the normal microbial population of healthy humans, is an opportunistic pathogen that can cause serious disease in immunocompromised patients. An important virulence factor of *C. albicans* is the formation of biofilms. These organized communities of cells are efficient at attaching to host cells and implanted medical devices. Carbonate has been studied as an agricultural antifungal agent, and here we demonstrate that carbonate can affect filamentation, biofilm formation, and antifungal drug resistance in *C. albicans*.

## Graphical abstract

Carbonate can reduce *Candida albicans* filamentation and biofilm formation in certain media, and it enhances sensitivity to some antifungal drugs.

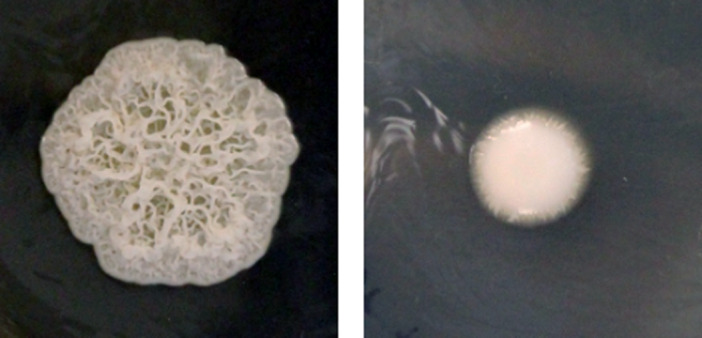

## 1 Introduction

Fungi are important causes of disease in both humans and agriculturally important plants (Steinberg and Gurr 2020). Mutations in *Aspergillus fumigatus* that resulted in resistance to field azole treatments also conferred resistance to azoles used in clinical settings and it is therefore of concern that agricultural use could be another potential source of antifungal resistance in human mycoses (Berger et al. 2017; Wiederhold 2017). In addition to developing new antifungals, there has been interest in using carbonate salts to treat diverse plant mycoses including apple scab (*Venturia inaequalis*) (Jamar, Lefrancq, and Lateur 2007), cucurbit foliar diseases such as powdery mildew (*Sphaerotheca fuliginea*) (Ziv 1992) and roses black spot (*Diplocarpon rosae*) (Horst 1992). There are examples of such compounds that are commercially available and used in home gardening (Wenneker 2016).

In contrast to the agricultural setting, there has been little investigation of carbonate salts on medically relevant fungi. For the most common human fungus, *Candida albicans*, work with carbonate has examined two mycosis scenarios. A 5% sodium bicarbonate solution reduced adhesion of *C. albicans* cells to denture material (acrylic resin) (Sousa et al. 2009) and elevated carbonate levels inhibited the growth of clinical isolates from foot onychomycosis or cutaneous fungal infection (Letscher‐Bru et al. 2013). Growth of *C. albicans* on implanted medical devices, such as vascular catheters, pacemakers, and artificial heart valves, is a serious problem (Kojic and Darouiche 2004; Hashemi Fesharaki et al. 2018; Glowacki, Quraishi, and Zakhireh 1990) and additional options for preventing or reducing growth on these surfaces are required. One of the challenges of treating *Candida* infections is the development of drug resistance (Wiederhold 2017; Baillie and Douglas 1999a; Ramage et al. 2005; Baillie and Douglas 1998, 1999b) and there is interest in chemicals that could be used in combination with existing drugs for a more effective treatment (Kim and Kim 2021). Chemicals that affect filamentation, the change in shape from round yeast cells to extended filaments, or biofilm formation have shown some promise in treating infections in animal models (Romo et al. 2018). *C. albicans* can grow on living and nonliving surfaces and in different host environments so we wanted to examine the effects of carbonate in a variety of conditions and growth media. Here we present results showing that carbonate can reduce filamentous growth and biofilm formation in some conditions and can alter resistance to antifungal drugs.

## 2 Materials and Methods

### 2.1 Strains and Media

The wild‐type strain SC5314 (Gillum, Tsay, and Kirsch 1984) was routinely maintained as a frozen −80℃ stock and grown on yeast extract‐peptone‐dextrose (YPD) agar.

### 2.2 Morphology Assays

Cells were grown overnight shaking in YPD 28℃ and then washed in sterile PBS. For liquid culture assays cells were diluted 1/20 into fresh prewarmed medium buffered to pH 7 using MOPS+/− 1% carbonate and grown shaking for at 37℃ for 4 h. Cells were then removed and photographed. For solid medium assays cells from overnight cultures were counted using a haemocytometer after washing with PBS, and resuspended to a final concentration of 5 × 10^6^ CFU/mL. Aliquots of 2 μL (1 × 10^4^ CFU) were spotted onto agar, incubated at 37℃ for 6 days and then photographed. To assess invasion of the medium, plates were washed gently under running water to remove loosely attached material and photographed again. Assays were done in biological triplicate.

### 2.3 Biofilm Formation

Biofilm formation was assessed using the 96‐well plate model described in (Pierce et al. 2008). Briefly, cells from an overnight culture were washed, counted and resuspended to a final concentration of 1 × 10^6^ CFU/mL in Spider (Liu, Köhler, and Fink 1994), GlcNAc (Hubbard, Sullivan, and Shepherd 1985) or YPD medium buffered to pH 7 using MOPS+/− 1% carbonate. Aliquots of 100 μL were used to seed wells in the 96‐well plate. Plates were incubated for 24 h at 37℃, washed to remove non‐adherent cells and stained with crystal violet. Biofilms were washed with sterile water to remove excess stain, then destained with 33% acetic acid (O'Toole 2011). The supernatant was transferred to empty wells and the OD_550_ was measured using a plate reader (BioTek). The results were analyzed by Student's *t*‐test, comparing each transformant strain with its parent strain. Biofilms were photographed using an inverted microscope before and after washing away non‐adherent cells.

### 2.4 Antifungal Sensitivity Testing

Antifungal sensitivity testing was performed following guidelines from the Centers for Disease Control (Antifungal Susceptibility Testing of Yeasts using Gradient Diffusion Strips. In: Antifungal Susceptibility Testing of Yeasts using Gradient Diffusion Strips 2024). Briefly, cells from an overnight culture were washed, counted and resuspended to a final concentration of 1 × 10^6^ CFU/mL. Cells were transferred to YPD plates buffered to pH 7 using MOPS+/− 1% carbonate with a sterile swab to make a lawn. Antifungal test strips (Liofilchem) were applied to the plates according to the manufacturer's instructions and the plates were incubated at 37℃ overnight before scoring.

## 3 Results and Discussion

### 3.1 Liquid Filamentation and Biofilm Assays

**Figure 1:**
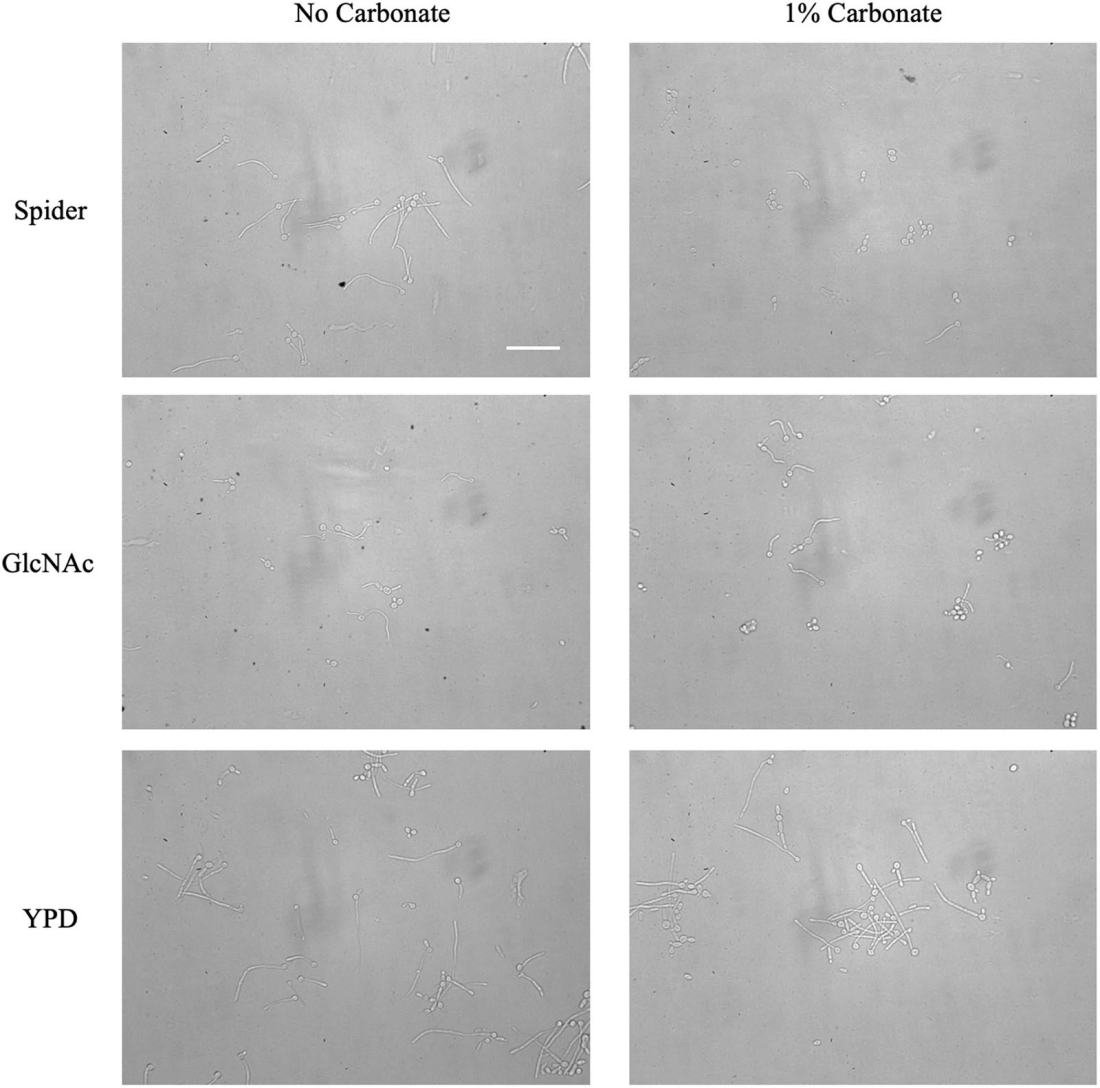
Liquid cultures. Strains were grown overnight at 28℃ then diluted 1/20 in prewarmed media buffered to pH 7 with MOPS+/− 1% carbonate and grown shaking at 37℃ for 4 h. Carbonate reduced filamentation in Spider but not the other media. The scale bar represents 50 microns.

**Figure 2:**
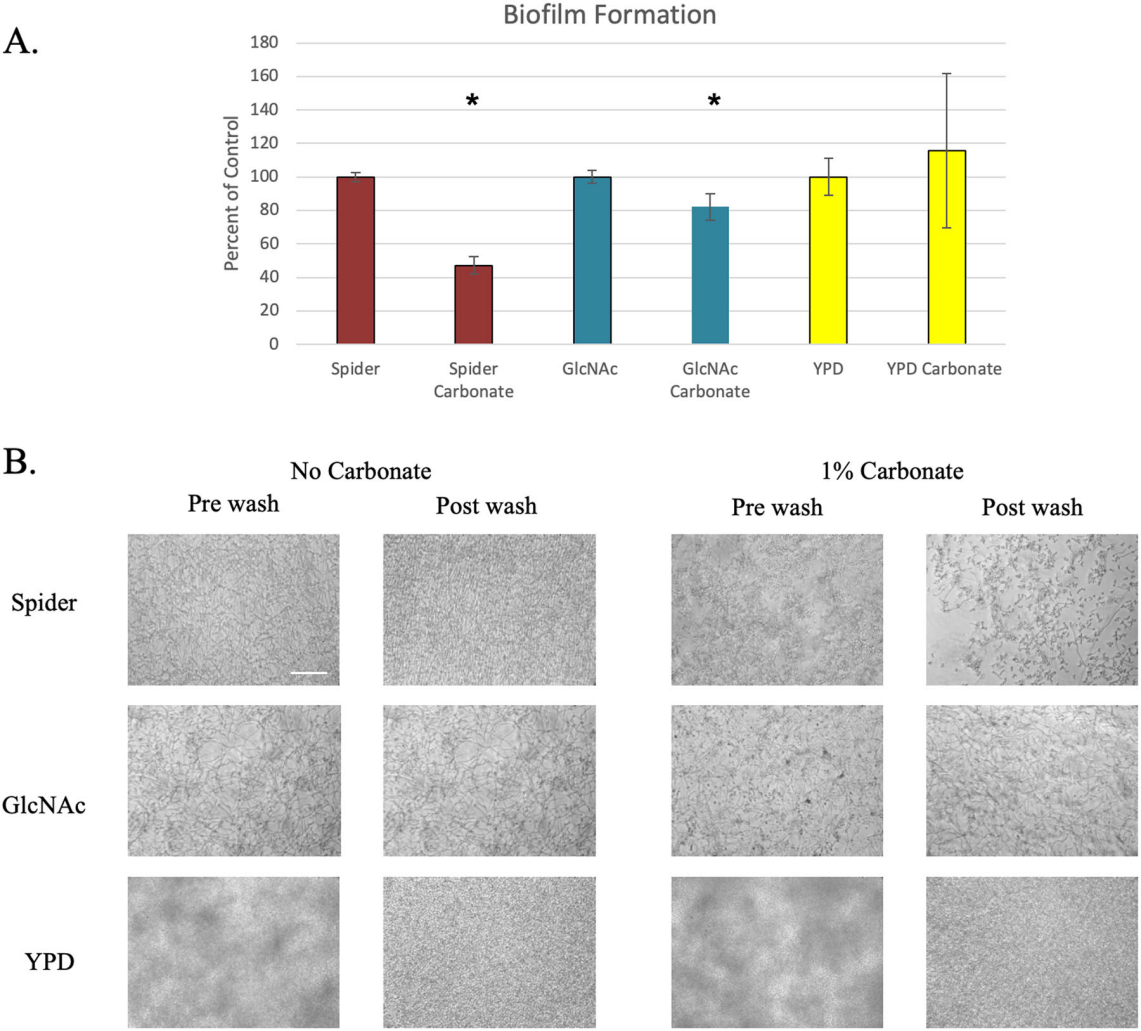
Biofilm formation. Biofilm formation was assessed using the 96‐well plate model. (A) Results were quantified by crystal violet staining. Biofilm formation is expressed as a percentage of the no carbonate medium, which is set to 100%, and the medium is indicated by color. Red, Spider; Blue, GlcNAc; Yellow YPD. Asterisks indicate statistically significant differences between the transformant and the parent strain. (B) Biofilms were photographed before and after washing away non‐adherent cells before staining. The scale bar represents 50 microns.

To examine the effects of carbonate on hyphal induction we grew cells in medium with and without 1% sodium carbonate. To maintain a consistent pH all media were buffered with MOPS to pH7. In Spider, GlcNAc and YPD the cells readily formed hyphae after a few hours (Figure 1). The presence of carbonate had no effect in YPD or GlcNAc, but strongly impaired hyphal growth in Spider medium. When biofilm formation was tested using the same media, a similar pattern was seen with significant reductions in biofilm formation in Spider and GlcNAc media with carbonate compared to without carbonate, but no difference in YPD (Figure 2A). The presence of carbonate did not impair the growth of the cells, but did, particularly in Spider medium, appear to affect filamentation (Figure 2B). This suggests that the effect of carbonate on cells grown in liquid media is to specifically inhibit some aspect of hyphal induction, rather than being a universal block. Different hypha‐inducing signals are detected by different sensors and may activate different pathways (Sudbery 2011). This result suggests carbonate may be acting on the cAMP‐PKA induction pathway.

### 3.2 Growth on Solid Media

**Figure 3:**
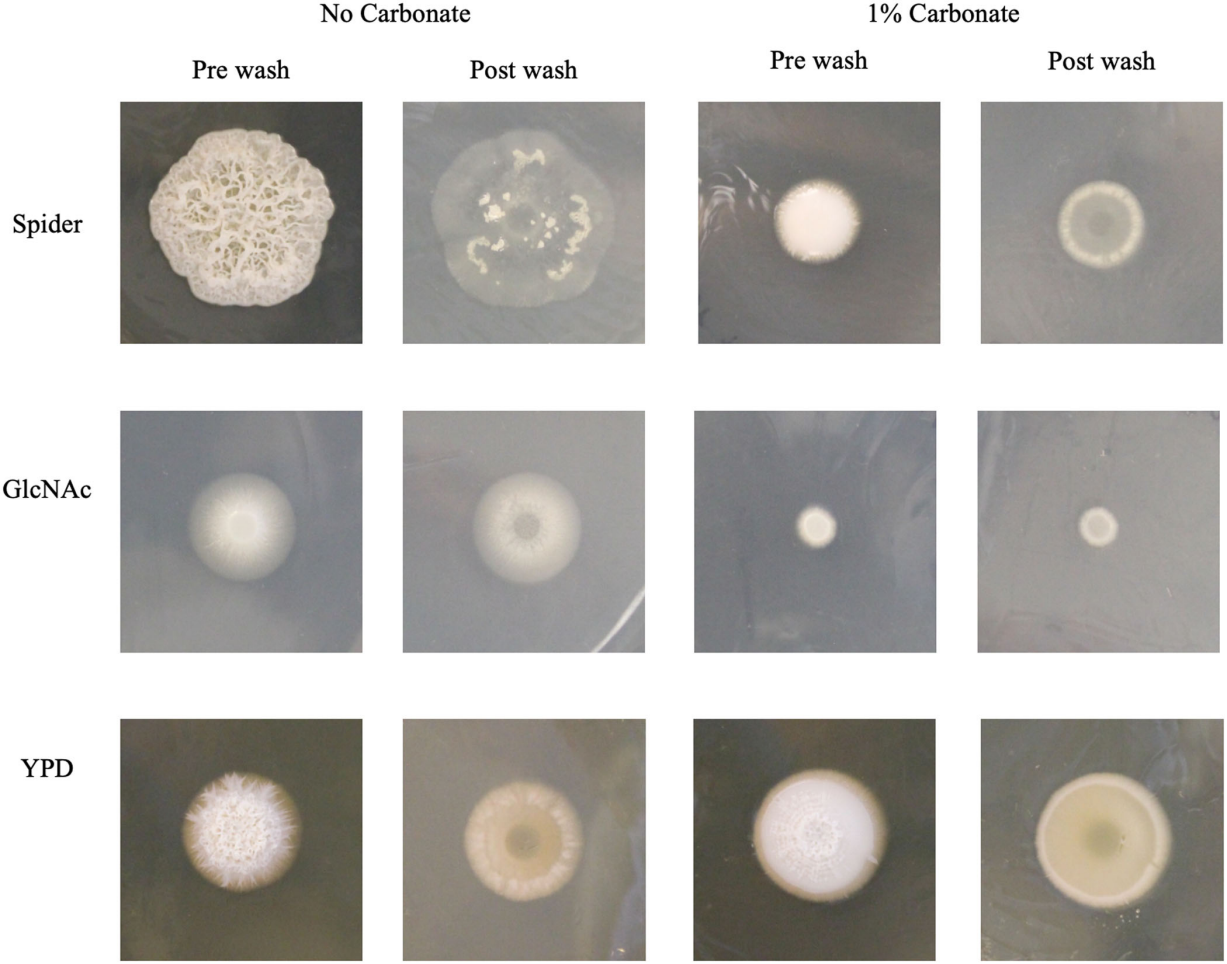
Morphology and Invasion of Solid Media. Cells were grown overnight at 28°C and then spotted onto solid media buffered to pH 7 with MOPS+/− 1% carbonate. Plates were incubated at 37℃ for 6 days and photographed before (prewash) and after (post‐wash) rinsing gently with running water to remove non‐adherent growth. Carbonate reduced colony wrinkling and the extent of invasion in all three media.

To examine the effects of carbonate on filamentation and on invasion of solid media, cells were spotted onto plates and after incubation surface growth was rinsed away to reveal invasive growth. As with the liquid culture assays, media were buffered with MOPS and growth was examined with and without 1% carbonate. On all three media tested (Spider, GlcNAc and YPD) colonies formed without carbonate were highly wrinkled whereas wrinkling was much reduced on the plates containing carbonate (Figure 3A). There was also a difference in peripheral invasive growth which was particularly extensive on GlcNAc but much more limited on GlcNAc with carbonate. Washing away surface growth revealed that the invasive growth in all three carbonate‐containing media was less extensive but also that the peripheral growth appeared to be denser than the plates without carbonate, with a bright white ring around the edge of the growth (Figure 3B). Unlike the effects in liquid media, it appears that the inhibition of wrinkling and extensive invasive growth was present across the different media. It has previously been observed that even in the same medium, the genetic control of hypha formation can be different between liquid and solid medium (Lee et al. 2023).

### 3.3 Antifungal Sensitivity

**Figure 4:**
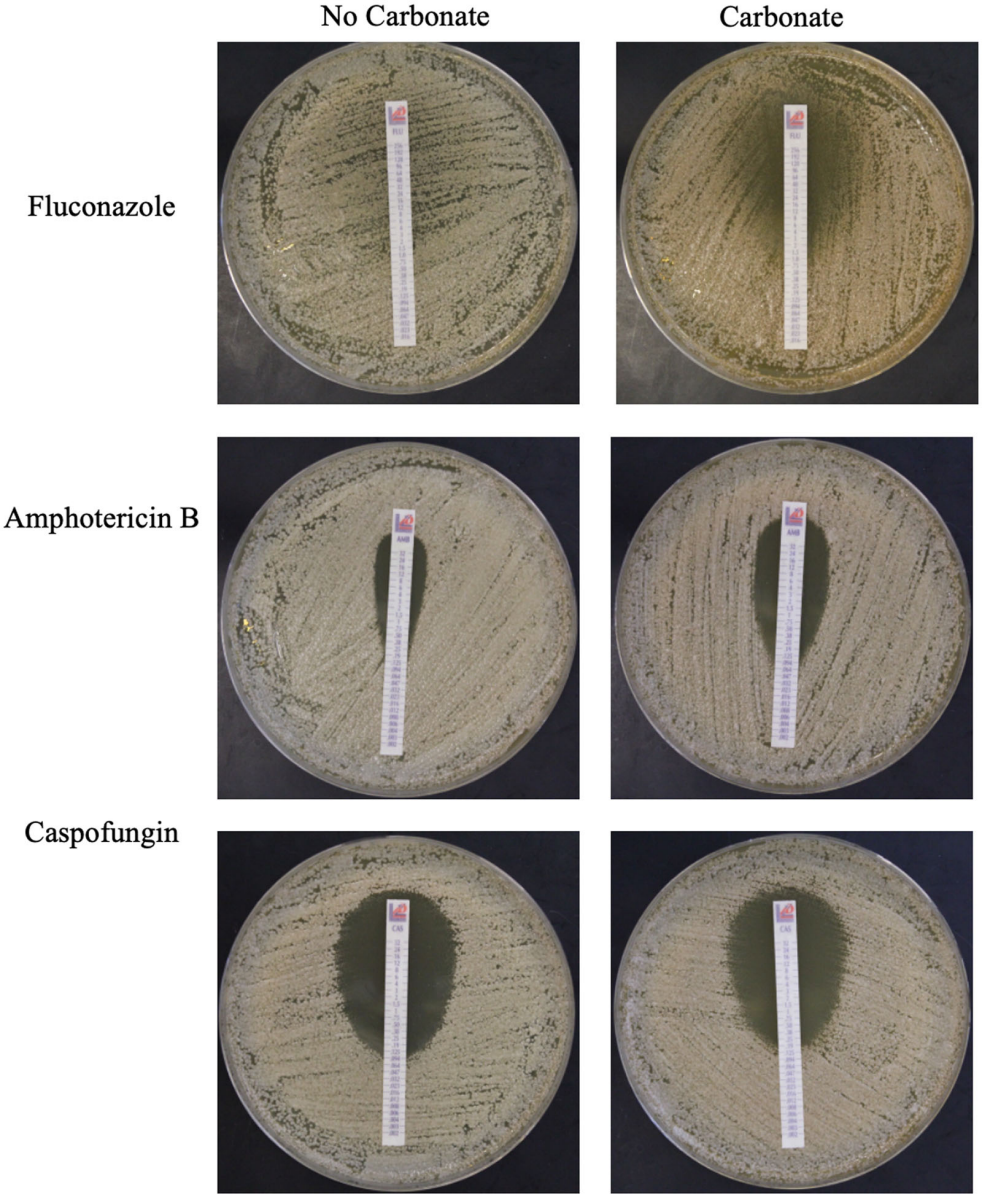
Antifungal Drug Resistance. Cells were grown overnight at 28℃ then spread on YPD plates buffered to pH7 with MOPS, +/− 1% carbonate to form a lawn. Antifungal test strips were applied, and plates were incubated overnight at 37℃.

Antifungal test strips were used to examine whether carbonate influenced sensitivity to different antifungal drugs (Figure 4). To examine drugs with different modes of action we used fluconazole, amphotericin and caspofungin strips. For fluconazole, without carbonate, there was no clearing, but there was inhibition to 1μg/ml. The addition of carbonate improved the clearing (to 4 μg/mL) but did not have much effect on the zone of inhibition (0.75 μg/mL). This is consistent with a synergy observed between thiabendazole and carbonate to control agricultural citrus decay (Schirra et al. 2008). For amphotericin B the MIC without carbonate was 0.19 μg/mL and with carbonate, it decreased to 0.064 μg/mL. For caspofungin, the MIC without carbonate was 0.094 μg/ml but with carbonate, it increased to 0.125 μg/mL. While we don't know the mechanism by which carbonate is influencing antifungal sensitivity, carbonate may be acting as a mild cell wall stressor and stimulating changes in cell wall architecture. Caspofungin inhibits the synthesis of β−1,3‐glucans, leading to weaker cell wall structure. Caspofungin exposure also results in increased chitin content and changes in the cell surface protein complement (Walker and Munro 2020). Perhaps cell wall changes induced by carbonate are strengthening the cell wall and lessening the impact of caspofungin or perhaps the changes are reducing access of caspofungin to the cell. Fluconazole inhibits synthesis of the membrane lipid ergosterol while amphotericin B associated with ergosterol and forms pores in the cell membrane. Carbonate improved the effectiveness of both of these compounds. Since they act by different mechanisms the presence of carbonate is likely having an effect on the cell rather than enhancing the specific action of both compounds.

These results indicate that carbonate can inhibit key virulence traits of filamentation and biofilm formation in some conditions, can reduce the extent of invasive growth, and can both increase and decrease antifungal MIC depending on the compound and its mode of action. Whilst carbonate salts are not likely to become an internal patient treatment, they could perhaps be useful in vitro or as a surface treatment to impair *C. albicans* biofilm development. Our results also reinforce the utility of searching for chemicals that may affect virulence traits without blocking growth wholesale.

## Author Contributions

**Trent Miedema:** conceptualization (supporting), investigation (equal), formal analysis (equal), writing–review and editing (equal). **Kayla Grooters:** conceptualization (lead), investigation (equal) formal analysis (equal), writing–review and editing (equal). **Ian Cleary:** conceptualization (supporting), formal analysis (supporting), supervision (lead), resources (lead), writing–original draft (lead).

## Ethics Statement

The authors have nothing to report.

## Conflicts of Interest

The authors declare no conflicts of interest.

## Data Availability

All data is provided in full in the results section of this paper.
